# Combined Targeting of BRAF and CRAF or BRAF and PI3K Effector Pathways Is Required for Efficacy in NRAS Mutant Tumors

**DOI:** 10.1371/journal.pone.0005717

**Published:** 2009-05-27

**Authors:** Bijay S. Jaiswal, Vasantharajan Janakiraman, Noelyn M. Kljavin, Jeffrey Eastham-Anderson, James E. Cupp, Yuxin Liang, David P. Davis, Klaus P. Hoeflich, Somasekar Seshagiri

**Affiliations:** 1 Department of Molecular Biology, Genentech Inc., South San Francisco, California, United States of America; 2 Department of Pathology, Genentech Inc., South San Francisco, California, United States of America; 3 Department of Immunology, Genentech Inc., South San Francisco, California, United States of America; 4 Department of Translational Oncology, Genentech Inc., South San Francisco, California, United States of America; City of Hope Medical Center, United States of America

## Abstract

**Background:**

Oncogenic RAS is a highly validated cancer target. Attempts at targeting RAS directly have so far not succeeded in the clinic. Understanding downstream RAS-effectors that mediate oncogenesis in a RAS mutant setting will help tailor treatments that use RAS-effector inhibitors either alone or in combination to target RAS-driven tumors.

**Methodology/Principal Findings:**

In this study, we have investigated the sufficiency of targeting RAS-effectors, RAF, MEK and PI3-Kinase either alone or in combination in RAS mutant lines, using an inducible shRNA *in vivo* mouse model system. We find that in colon cancer cells harboring a KRAS^G13D^ mutant allele, knocking down KRAS alone or the RAFs in combination or the RAF effectors, MEK1 and MEK2, together is effective in delaying tumor growth *in vivo*. In melanoma cells harboring an NRAS^Q61L^ or NRAS^Q61K^ mutant allele, we find that targeting NRAS alone or both BRAF and CRAF in combination or both BRAF and PIK3CA together showed efficacy.

**Conclusion/Significance:**

Our data indicates that targeting oncogenic NRAS-driven melanomas require decrease in both pERK and pAKT downstream of RAS-effectors for efficacy. This can be achieved by either targeting both BRAF and CRAF or BRAF and PIK3CA simultaneously in NRAS mutant tumor cells.

## Introduction

Oncogenic mutations in the RAS family of small GTPases, KRAS, HRAS and NRAS, occur in approximately a third of all human cancers [1]–[3]. This makes RAS a prime target for drug development. However, efforts at developing therapeutics that target mutant RAS directly so far have not been very successful [2].

Understanding the signaling pathways engaged by oncogenic RAS in promoting malignant transformation is fundamental to identifying and targeting components downstream of RAS. Several studies have helped identify major RAS effector molecules which include the Raf kinases, class I phosphoinositide 3-kinases (PI3K), Ral guanine nucleotide exchange factors (Ral-GEFs), Rac exchange factor Tiam1, and phospholipase Cε [4], [5].

The RAF kinases, members of the three-component MAP Kinase cascade, once activated by GTP-bound RAS, phosphorylate and activate dual-specificity kinases MEK1 and MEK2, which in-turn activate MAP Kinases ERK1 and ERK2. Active ERK1/2 then translocates to the nucleus to exert their biological effects. PIK3CA, a PI3K family member stimulated through RAS activation, phosphorylates its lipid substrate phosphatidylinositol (4,5) bisphosphate (PIP2) to produce phosphatidylinositol (3,4,5) triphosphate (PIP3). This phosphorylated lipid PIP3 is an important cellular second messenger that promotes the activation of AKT (AKT1, AKT2 and AKT3) leading to cell survival.

Besides mutations in RAS, activating somatic mutations in its effectors PIK3CA and BRAF, occur in various human cancers [6], [7]. While PIK3CA mutations are more prevalent in colon and breast cancer, BRAF mutation occurs at a high frequency in melanoma [6], [7]. Additionally, loss-of-function of the PTEN tumor suppressor gene, which leads to activation of PI3-kinase, is widespread in cancers [8]. Functional studies that dissect PIK3CA and BRAF somatic mutations have established the oncogenic nature of these RAS effector genes [9]–[11]. Further, these studies show that each effector arm on its own when activated is sufficient to promote tumor formation, especially, in tumor types where they are commonly mutated. Interestingly, BRAF mutations are mutually exclusive with NRAS mutations in melanoma and KRAS mutations in colorectal cancer [7], [12], [13]. In contrast, mutations in PIK3CA are not mutually exclusive with KRAS in colorectal cancers [12], [13]. The patterns of mutational co-occurrence, suggests that each RAS-effector is differentially employed in promoting tumor initiation in a tumor tissue specific manner. A recent study using an engineered mouse model showed the importance of intact signaling via the PI3K RAS-effector arm for initiation of lung tumors in a RAS-mutant mouse [14]. Similarly, in engineered human skin graft mouse models, the PI3K effector arm was found to be equivalent to RAS in inducing melanocytic neoplasia [15]. Another study, using engineered cell lines that express various RAS-mutants that can preferentially engage either RAF, PI3K or RalGEF showed that while all three were important for tumor initiation, the PI3K pathway was essential for tumor maintenance [16].

Pharmacological inhibitors that target RAS effectors, RAF and PI3Ks, are in various stages of clinical trials [17], [18]. Small molecule inhibitors that target downstream components of these effectors, MEK1/2 and AKT1/2/3 have been developed and are undergoing tests in human trials as well [18]–[21]. Using pharmacological inhibitors of PI3K and RAF effectors, various studies have demonstrated the importance of either the PI3K or RAF arms or the requirement for targeting both in RAS mutated tumors [22]–[25]. In pancreatic epithelial cells, KRAS mediated transformation requires both RAF and PI3K signaling [26]. In NRAS mutated melanoma cells, CRAF was demonstrated to be the major RAS effector for signaling through ERK [27], [28]. These along with the studies using engineered mouse models and cell lines have yielded results that are often contradictory and confounding, underscoring the importance of further *in vivo* studies that address the relevance of these components in initiation, maintenance and progression of cancers.

Using a recently described inducible shRNA system [29], we have previously shown that signaling via oncogenic BRAF is essential for tumor initiation and maintenance in melanoma models [30]. In this study, we have used this inducible shRNA system and *in vivo* xenograft mouse model to demonstrate the effectiveness of targeting downstream RAS-effectors either alone or in combination as strategies for treatment of RAS driven cancers.

## Results

### Oncogenic RAS-mutant cancer cells require RAS for proliferation, anchorage independent growth and tumor formation

**Figure 1:**
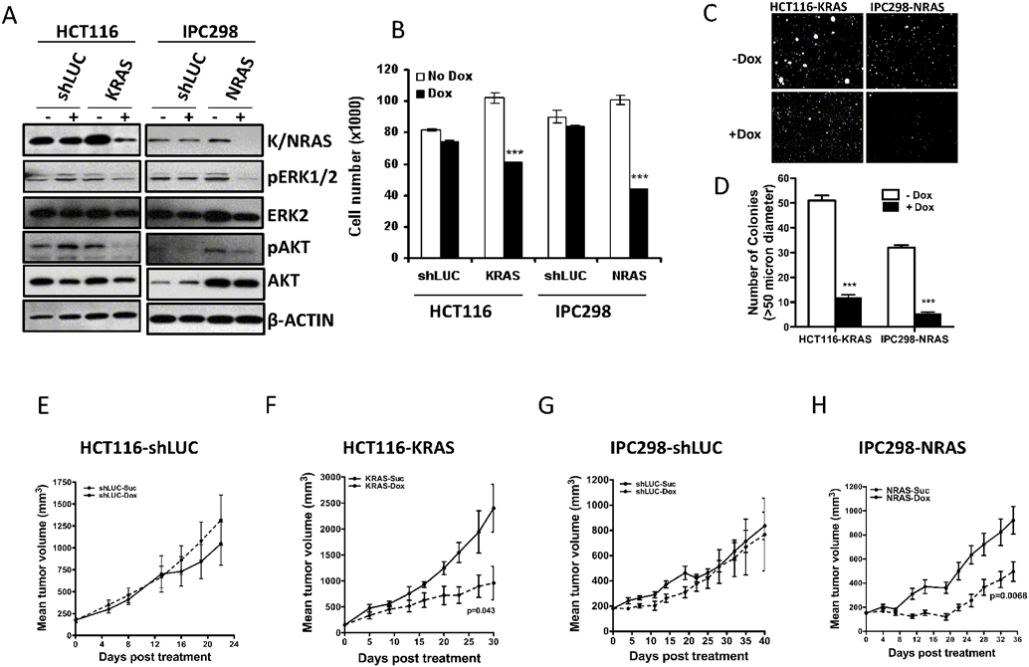
KRAS or NRAS, in RAS mutant lines, is required for cell proliferation, anchorage independent growth and *in vivo* tumor growth and progression. (A) Western blot analysis of KRAS and NRAS knock-down in HCT116 and IPC298 cells, respectively. Lysates from cells expressing KRAS, NRAS or control luciferase (shLUC) shRNA, prepared 72 h post treatment with dox, were analyzed, as indicated, by immunoblotting. β-actin levels in the blot serves as a loading control. (B) Proliferation of KRAS or NRAS shRNA expressing cells after 4 days post dox treatment. (C–D) KRAS and NRAS shRNA induction, in HCT116 and ICP298 respectively leads to reduced anchorage independent growth of these cells. Representative images (C) and colony count (D) are shown. (E–H) RAS knock-down, KRAS in HCT116 (F), and, NRAS in IPC298 (H), delays *in vivo* tumor growth. Induction of LUC shRNA in control cells (E, G) had no effect on tumor growth. Each data point is the mean±SEM tumor volume derived from 10 mice. Dotted line in (E–H) represents data from dox treated animals.

HCT116, a colon cancer line that harbors a KRAS^G13D^ mutation and IPC298, a cutaneous melanoma cell line bearing an NRAS^Q61L^ mutation, were chosen for this study since KRAS in colorectal cancer and NRAS in melanoma is the most frequently mutated RAS gene in these tumor types. The colon cancer cell line HCT116, in addition to KRAS mutation, also harbors an activating mutation in the RAS effector PIK3CA (Table S1). In order to understand the relevance of oncogenic RAS and its dependence on its major downstream effectors, RAF and PI3K, we used a previously described doxycycline (dox)-inducible shRNA system [29] to study the effects of RAS knockdown on cellular proliferation and tumor growth. We generated pools of cells expressing shRNAs that target RAS in a dox-inducible fashion. Upon dox treatment RAS, KRAS in HCT116 and NRAS in IPC298, was effectively silenced (Figure 1A). Consistent with the loss of signaling from RAS, the levels of phospho ERK decreased in both the RAS mutant lines, relative to the total ERK in these lines. In addition to loss of phosphoERK levels, RAS knock-down in both IPC298 and HCT116 cells led to a decrease in the phospho AKT levels. Phospho AKT levels were reduced in KRAS knock-down HCT116 cells despite the presence of PIK3CA mutation in this line. In both lines the control luciferase shRNA-expressing cells upon dox induction did not show any changes in either the phospho or the total ERK levels. Similarly, the phospho and total AKT levels were not modulated significantly in these cells following dox addition. To determine the requirement of RAS in these RAS-mutant cancer lines for cellular proliferation, we studied these lines for growth following induction of shRNAs that target NRAS or KRAS. We found that, consistent with the loss of downstream signaling following abrogation of KRAS in HCT116 and NRAS in IPC298, cell proliferation was reduced by 40% and 60% respectively (Figure 1B). We found a similar trend in proliferation with a second shRNA that targeted KRAS or NRAS in these lines (data not shown). Compared to the RAS knock-down lines, the luciferase control lines showed no effect on proliferation following dox induction (Figure 1B). In addition to the effect seen on proliferation, RAS knock down led to a 5-6-fold decrease in anchorage-independent growth in both the HCT116 and IPC298 cells (Figure 1C and 1D). These results demonstrate that both lines were dependent on RAS, KRAS in HCT116 and NRAS in IPC298, for their proliferation and anchorage-independent growth. We next tested the relevance of oncogenic RAS in the RAS-mutant lines for tumor formation *in vivo* by establishing s.c. tumors and inducing the targeting shRNA in established tumors as described in materials and methods. As shown in Figure 1E and G, the control luciferase oligo induction had no effect on the growth of HCT116 or IPC298 *in vivo*. However, knocking down KRAS in HCT116 (Figure 1F) and NRAS in IPC298 (Figure 1H) led to a significant delay in tumor growth, indicating the requirement of KRAS in HCT116 and NRAS in IPC298 for tumor growth *in vivo*.

### Differential effects of targeting downstream RAS-effectors on tumor growth in RAS mutant cancer cells

RAS predominantly engages the RAF pathway and the PI3K pathway as major downstream effectors in various cancers. We sought to examine the contribution of these two arms in tumor growth and progression. In addition to elucidating the role of these two RAS-effector arms in cancer, this knowledge should provide insight into targeting RAS-driven tumors. We therefore generated pools of either HCT116 or IPC298 cells harboring inducible shRNAs that target, BRAF, CRAF, MEK1, MEK2 and PIK3CA either singly or in various combinations within the same line. We then tested these lines for *in vitro* proliferation and *in vivo* tumor growth.

**Figure 2:**
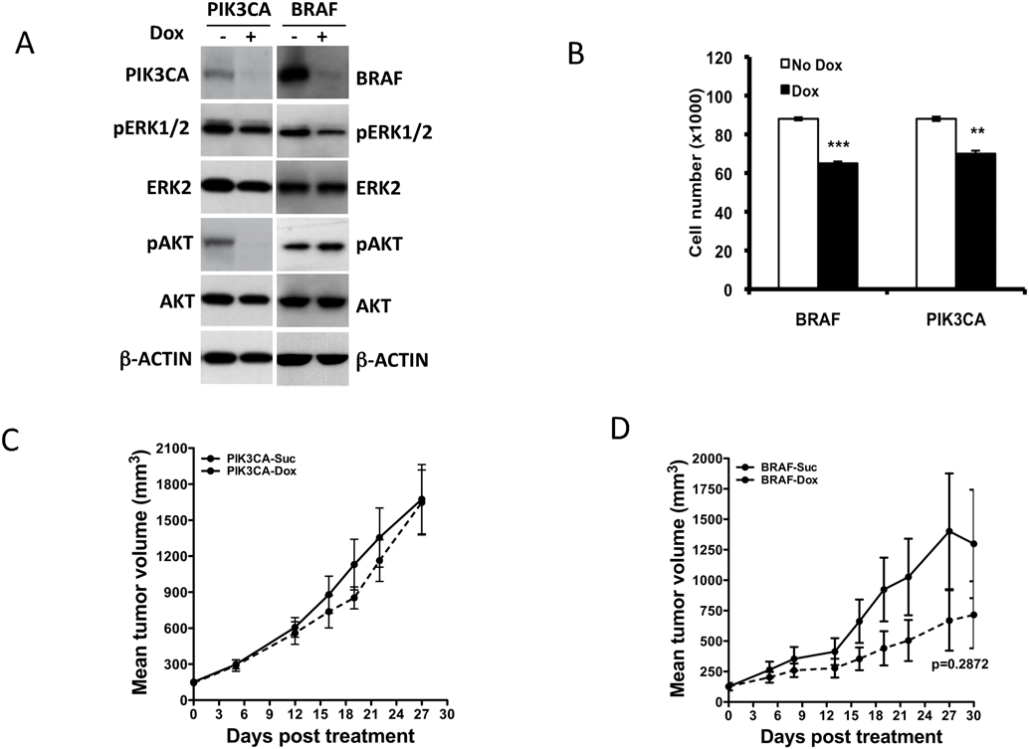
Effect of BRAF and PIK3CA knock-down on KRAS^G13D^ mutant HCT116 cell growth. (A) Western blot analysis of PIK3CA or BRAF knock-down in HCT116 at 72 h post dox induction of relevant shRNAs. The effect of knock-down on the phosphorylation status of relevant downstream targets is shown. (B) Proliferation of BRAF and PIK3CA shRNA expressing cells 4 days post dox treatment. (C) shRNA targeting PIK3CA when induced in mice bearing HCT116 tumors did not delay tumor growth. (D) BRAF knock-down in HCT116 derived tumors shows a trend towards delayed tumor growth. Each data point is the mean±SEM tumor volume derived from 10 mice. Dotted line in (C, D) represents data from dox treated animals.

**Figure 3:**
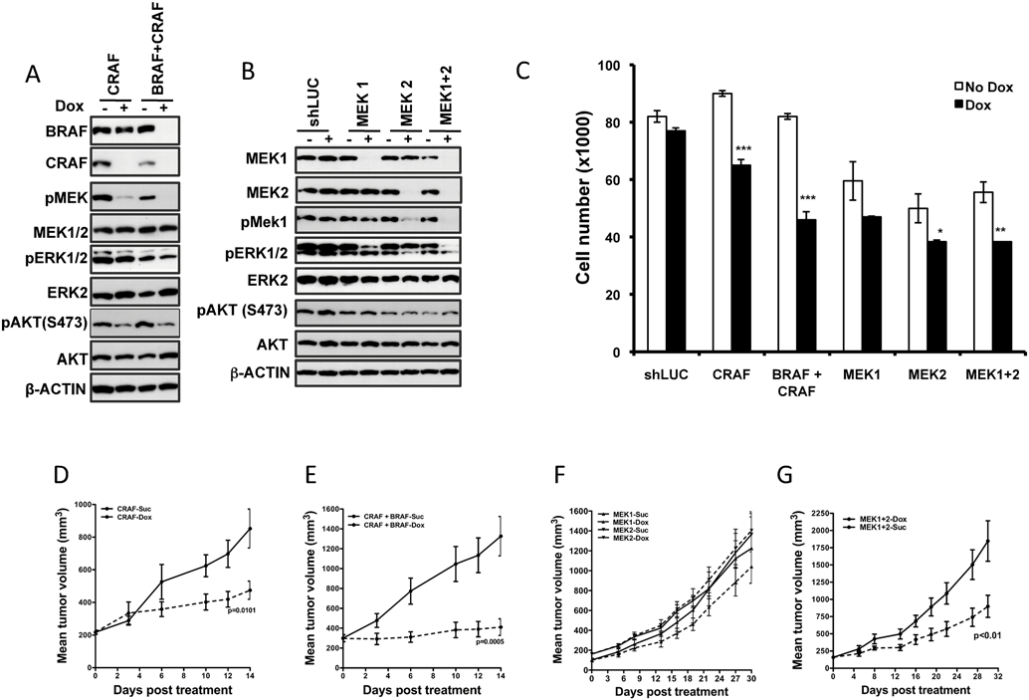
RAF or MEK1-MEK2 double knock-down delays growth of KRAS^G13D^ mutant HCT116 cells *in vivo*. (A–B) Western blot analysis of CRAF or CRAF and BRAF or MEK1 and MEK2 knock-down in HCT116 at 72 h post dox induction of relevant shRNAs. The effect of knock-down on the phosphorylation status of relevant downstream target is shown. (C) Proliferation of CRAF, CRAF and BRAF, MEK1, MEK2 and double MEK1+2 shRNA expressing cells 4 days post dox treatment. (D–G) shRNA targeting CRAF (D), CRAF and BRAF (E) and MEK1 and MEK2 double knock-down (G) in KRAS-mutant HCT116 tumors *in vivo* delayed tumor growth. shRNA targeting MEK1 or MEK2 individually (F) when induced in mice bearing HCT116 tumors did not delay tumor growth. Each data point is the mean±SEM tumor volume of 10 mice. Dotted line in (D–F) represents data from dox treated animals.

HCT116-BRAF knockdown line, upon dox treatment showed reduction in BRAF protein levels and a concomitant decrease in phospho ERK levels relative to the total ERK levels (Figure 2A). Similarly, HCT116-PIK3CA knockdown lines when treated with dox showed a reduction in the levels of PIK3CA and a decrease in phospho AKT compared to the control un-induced cells. In proliferation studies, both the dox treated PIK3CA knockdown and BRAF knockdown HCT116 lines showed a 20–25% reduction in proliferation compared to non-dox treated cells (Figure 2B). However, *in vivo*, mice bearing tumors derived from PIK3CA knock-down HCT116-cells, showed no effect on tumor growth following dox treatment (Figure 2C). These results are consistent with the fact that the mutant PIK3CA in HCT116 did not functionally substitute for KRAS in experiments where KRAS was silenced, both *in vitro* and *in vivo* (Figure 1B and 1F). In contrast to knock-down of PIK3CA in HCT116, nude mice bearing subcutaneous tumors resulting from injected HCT116 BRAF-inducible shRNAs cells, when treated with dox showed a delay in tumor growth (Figure 2D). The delay was not statistically significant, in part due to the outlier tumor volumes observed towards the end of this study. However, this could also be in part due to the fact that the oncogenic RAS could engage other RAF family members like CRAF and continue to promote tumor growth and survival. To fully evaluate the contributions of the RAF arm that could result from engagement of other RAFs, we generated HCT116 inducible shRNA knock-down lines where we conditionally silenced CRAF or both CRAF and BRAF together. As expected, knock-down of BRAF and CRAF together decreased pMEK level completely, while knock-down of CRAF alone showed some residual pMEK levels (Figure 3A). Consistent with this finding, the BRAF/CRAF double knock-down line showed a decrease in pERK levels while the CRAF knock-down alone did not substantially affect pERK levels *in vitro*. However, knock-down of either CRAF or BRAF and CRAF together decreased pAKT levels (Figure 3A). In proliferation studies, CRAF knock-down lines showed a ∼25% decrease in proliferation, while the combined CRAF and BRAF knock down had ∼40% reduction in proliferation (Figure 3C). I*n vivo*, combined CRAF and BRAF knock-down showed a significant delay in tumor growth compared to the un-induced control (Figure 3E). Although not as significant as the dual RAF knock-down, CRAF knock-down alone significantly delayed tumor growth (Figure 3D). These results suggest that KRAS in HCT116 cells engages both CRAF and BRAF for promoting tumor growth with CRAF being the dominant effector arm.

Given that several Mek inhibitors are in development in the clinic we sought to test if targeting the RAF effectors MEK1 or MEK2 either alone or together in the same pool of cells would be as effective as targeting the RAFs. We therefore generated HCT116 cells that inducibly express shRNAs targeting MEK1 or MEK2 or both. In these lines upon dox addition, MEK1 in the MEK1-knock-down line, MEK2 in the MEK2-knock-down line, and both MEK1 and MEK2 in the double knock-down line were decreased, compared to the un-induced control (Figure 3B). While the MEK1 or MEK2 knock-down alone did not show a substantial decrease in phospho ERK levels, the double knock-down showed a significant decrease in phopsho ERK levels (Figure 3B). However, knock-down of MEK either alone or in combination does not affect pAKT level (Figure 3B). We tested these lines *in vitro* for their ability to proliferate in the presence or absence of dox and found that the MEK1, MEK2 and MEK1+2 double lines showed ∼15%, ∼20% and ∼30% reduction in proliferation respectively, compared to untreated control cells (Figure 3C). This suggests that loss of each isoform of MEK has an effect on its own, while the combined knock-down has the most effect on proliferation. We tested the MEK-knock-down HCT116 lines *in vivo* for tumor growth in nude mice and found that silencing both MEK1 and MEK2 together led to a significant delay in tumor growth (Figure 3G), compared to targeting them individually (Figure 3F). To confirm this further we tested a pharmacological inhibitor of MEK on HCT116 cells and found that it is effective in delaying tumor growth (Figure S1). These results suggest that in the KRAS mutant HCT116 colon cancer cells, the RAF/MEK/ERK effector arm is necessary for cellular proliferation and tumor growth, despite the presence of activating mutation in PIK3CA.

**Figure 4:**
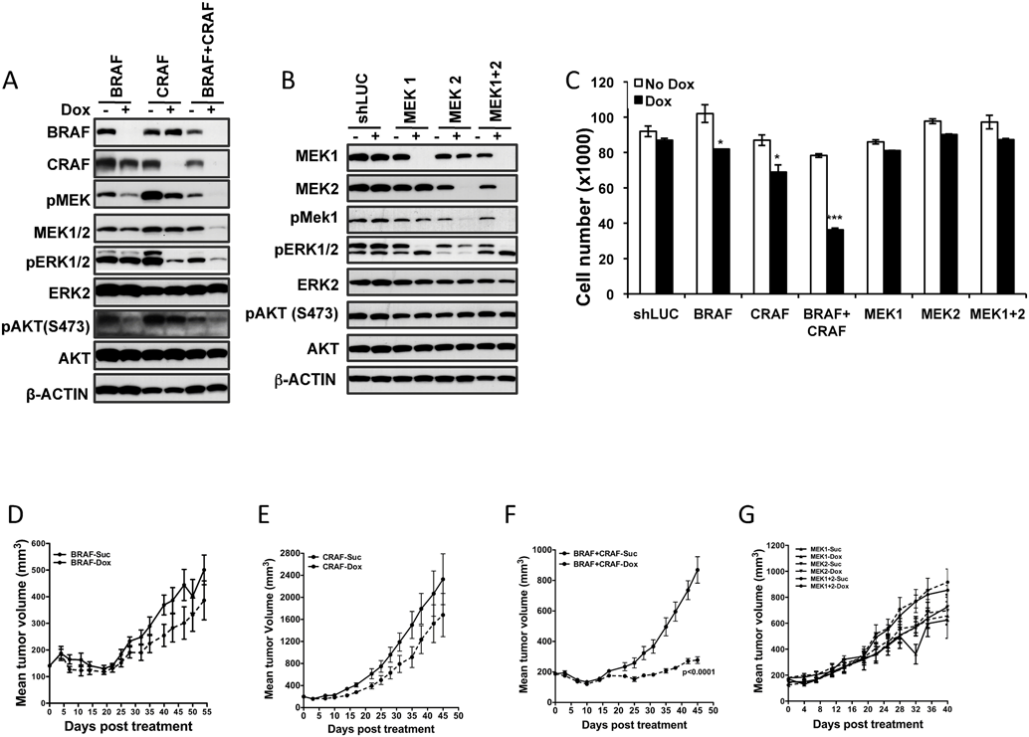
BRAF/CRAF double-knock-down delays growth of NRAS^Q61L^-mutant IPC298 melanoma cells *in vivo*. (A–B) Western blot analysis showing BRAF, CRAF, BRAF+CRAF, MEK1, MEK2, and double MEK1/2 knock-down in IPC298 at 72 h after dox induction of shRNAs. The effect of knock-down on the phosphorylation status of relevant downstream targets is shown. (C) Effect of expressing BRAF, CRAF, BRAF+CRAF, MEK1, MEK2 and double MEK1+2 shRNAs on proliferation of IPC298 cells 4 days following dox treatment. (D–G) While shRNA targeting BRAF+CRAF (F) together delayed tumor growth, shRNA targeting BRAF (D), CRAF (E), MEK1, or MEK2 or MEK1 and MEK2 together (G) when induced in mice bearing IPC298 derived tumors did not delay tumor growth. Each data point is the mean±SEM tumor volume of 10 mice. Dotted line in (D–G) represents data from dox treated animals.

We extended these studies to the NRAS mutant melanoma IPC298 cells. Given that BRAF is more commonly mutated in melanomas, we hypothesized that targeting the BRAF effector arm alone would be sufficient to affect cell proliferation and *in vivo* tumor growth. To test this, we generated a stable pool of IPC298 cells harboring an inducible BRAF shRNA. Upon BRAF shRNA induction, we observed a significant decrease in the BRAF protein (Figure 4A). *In vitro* the BRAF-shRNA expressing IPC298 cells showed a ∼20% decrease in proliferation upon dox treatment relative to the uninduced control cells (Figure 4C). However, *in vivo*, we found that BRAF silencing did not show a statistically significant delay in IPC298 tumor growth (Figure 4D). This is contrary to our initial hypothesis about BRAF playing a dominant role in tumor cell signaling in melanomas bearing an NRAS mutation. The lack of efficacy in IPC298 cells following BRAF knock-down could be the result of mutant RAS engaging other RAF family member like CRAF. To address this, we generated and tested IPC298 pools that inducibly expressed shRNAs targeting CRAF alone or BRAF and CRAF in combination. Upon induction of corresponding shRNA in these cells, CRAF or BRAF and CRAF proteins were completely depleted leading to a decrease in pMEK and pERK levels (Figure 4A). As with BRAF, knock-down of CRAF also decreased *in vitro* cell proliferation by ∼20%. We found double knock-down of both BRAF and CRAF to be far more effective leading to a ∼50% reduction in cell proliferation compared to the uninduced cells (Figure 4C). Similar to the effects observed with BRAF knock-down, silencing CRAF alone did not show a statistically significant delay in IPC298 tumor growth in vivo (Figure 4E). In contrast, mice bearing IPC298 co-expressing BRAF and CRAF shRNA showed complete inhibition of tumor growth following dox induction (Figure 4F). These data indicate that the NRAS mutant IPC298 melanoma line engages both the BRAF and CRAF effector arms for survival and proliferation and that targeting both BRAF and CRAF is important for efficacy. Further, both pERK and pAKT levels are decreased only when both BRAF and CRAF are knocked down together (Figure 4A), suggesting that signaling from both the RAF and PI3K effectors need to be inhibited for efficacy.

Since, both BRAF and CRAF signal through their effectors MEK1 and MEK2, we sought to understand the effect of targeting MEKs in IPC298 cells. We generated and tested IPC298 pools that inducibly expressed shRNAs that target RAF-effectors MEK1 or MEK2 or both MEK1 and MEK2 together in the same cells. The targeted knock-down of MEK1 or MEK2 alone or a combined MEK1 and MEK2 knock-down reduced the levels of these proteins (Figure 4B). The pERK levels were also decreased in these cells compared to control untreated cells (Figure 4B). In all the MEK-shRNA containing IPC298 lines, *in vitro* we did not observe a significant difference in proliferation between the control and dox-treated cells (Figure 4B). Similarly, silencing MEK1 or MEK 2 alone or in combination, did not show a significant delay in tumor growth relative to the control animals (Figure 4G). This result was unexpected given that CRAF and BRAF targeting was effective, suggesting potential differential survival and proliferation signaling outputs resulting from inhibition of different nodes along the RAS/RAF pathway. Consistent with this, we find pAKT levels to be unchanged (Figure 4B) in the MEK1/2 double knock-down line, although pERK was decreased. This is in contrast to decreases in both the pAKT and pERK levels observed with the BRAF/CRAF double knock-down lines (Figure 4A). Further, this is a likely reason for the lack of efficacy observed in the combined MEK1/2 knock-down as compared to BRAF/CRAF double knock-down. Although the exact cross talk and feedback mechanism leading to the differential modulation of pAKT and pERK in response to knock-down of the various RAF and RAF-effectors is not obvious, this data indicates the requirement for targeting signaling from both ERK and AKT effectors in NRAS mutant lines.

### Combined targeting of RAF and PI3K, RAS-effector arms effectively reduces tumor growth in NRAS mutant tumors

Given the observed requirement for decrease in both pERK and pAKT downstream of mutant NRAS and the intense efforts to develop inhibitors targeting BRAF, MEK1/2 and PI3K in progress, we sought to understand if combined knock-down of RAF or MEK with PI3K would be an effective strategy for treatment of NRAS mutant melanomas.

**Figure 5:**
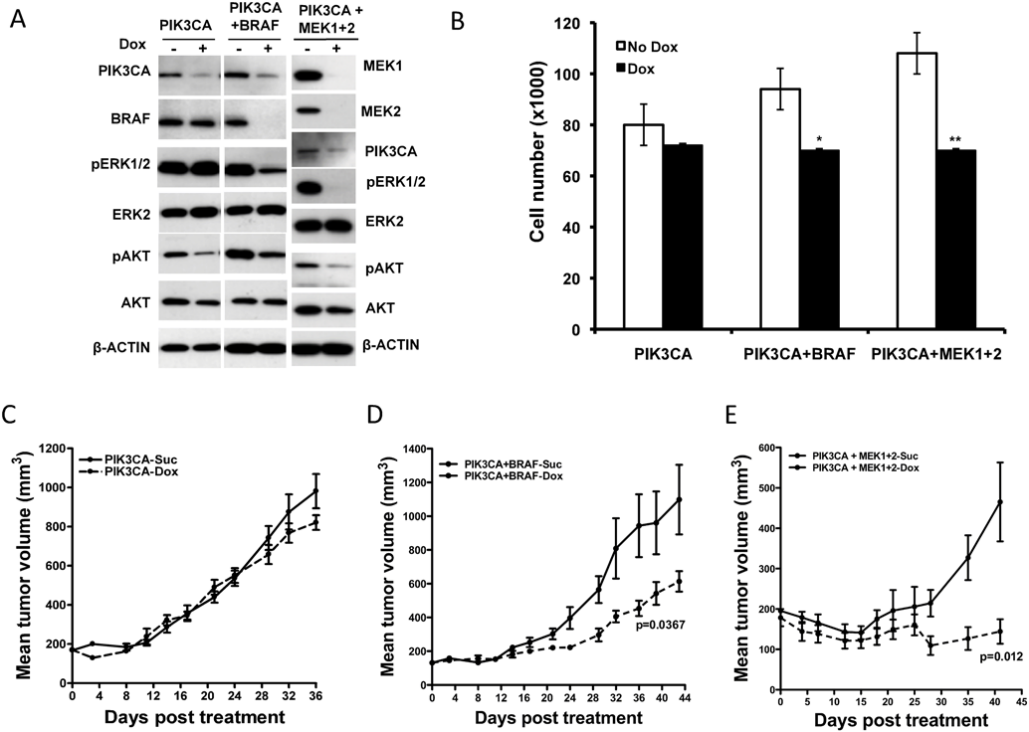
Combined targeting of PIK3CA+BRAF, and PIK3CA+MEK1/2 in NRAS^Q61L^-mutant IPC298 melanoma cell delays tumor growth *in vivo*. (A) Immuno blot analysis of PIK3CA, PIK3CA+BRAF, and PIK3CA+MEK1/2 knock-down in IPC298 cells affects phosphorylation of downstream targets. (B) Proliferation of IPC298 cells expressing shRNAs targeting PIK3CA alone or PIK3CA and BRAF together or PIK3CA together with MEK1/2 4 days after dox treatment. (C) shRNA targeting PIK3CA in IPC298 did not delay tumor growth *in vivo*, following induction of PIK3CA targeting shRNA. (D–E) Combined induction of BRAF and PIK3CA shRNA together or MEK1 and 2 together with PIK3CA in mice bearing IPC298 derived tumors delay tumor growth *in vivo*. Each data point is the mean±SEM tumor volume of 10 mice. Dotted line in (C–E) represents data from dox treated animals.

Induction of shRNA targeting PIK3CA alone reduced the levels of PIK3CA, leading to a reduction in the pAKT levels (Figure 5A). However, this did not decrease the pERK levels (Figure 5A). The PIK3CA shRNA line did not show any significant decrease in proliferation following dox treatment, compared to untreated cells (Figure 5B). *In vivo*, induction of PIK3CA targeting shRNA, did not delay IPC298 tumor growth, compared to animals that were placed on sucrose (Figure 5C). This suggested that targeting the PIK3CA effector arm alone was not sufficient to prevent tumor growth in the NRAS mutant line.

To test the possibility that the combined targeting of RAF and PI3K effector arms would be effective in the NRAS mutant background, we generated IPC298 pools that inducibly expressed shRNAs that target either PIK3CA and BRAF together or PIK3CA along with both the RAF-effectors, MEK1 and 2. These cells were characterized for effective knock-down of BRAF, MEKs and PIK3CA. As expected, upon dox treatment the cells showed reduced BRAF, PIK3CA or MEK1 and 2 protein (Figure 5A). Consistent with this, pERK and pAKT levels were diminished, relative to the total amount of ERK and AKT respectively. It is interesting to note that while BRAF or PIK3CA knock-down alone did not affect pERK levels significantly, the combined knock-down of BRAF and PIK3CA led to significant decrease in pERK and pAKT. We studied these cells in proliferation assays and found that there was a ∼25–30% decrease in proliferation when both BRAF and PIK3CA or the RAF-effectors MEK1, 2 and PIK3CA together were knocked down in the IPC298 cells (Figure 5B). We also tested these lines *in vivo* to see if targeting both BRAF and PIK3CA or both MEK1+2 and PIK3CA will lead to delayed tumor growth. Targeting both the RAS-effector arms, either by combined BRAF and PIK3CA or MEK1+2 and PIK3CA together, effectively delayed the tumor growth (Figure 5D and 5E) confirming the importance of both the PI3K and RAF effector arms in the IPC298 NRAS-mutant melanoma line for tumor growth.

**Figure 6:**
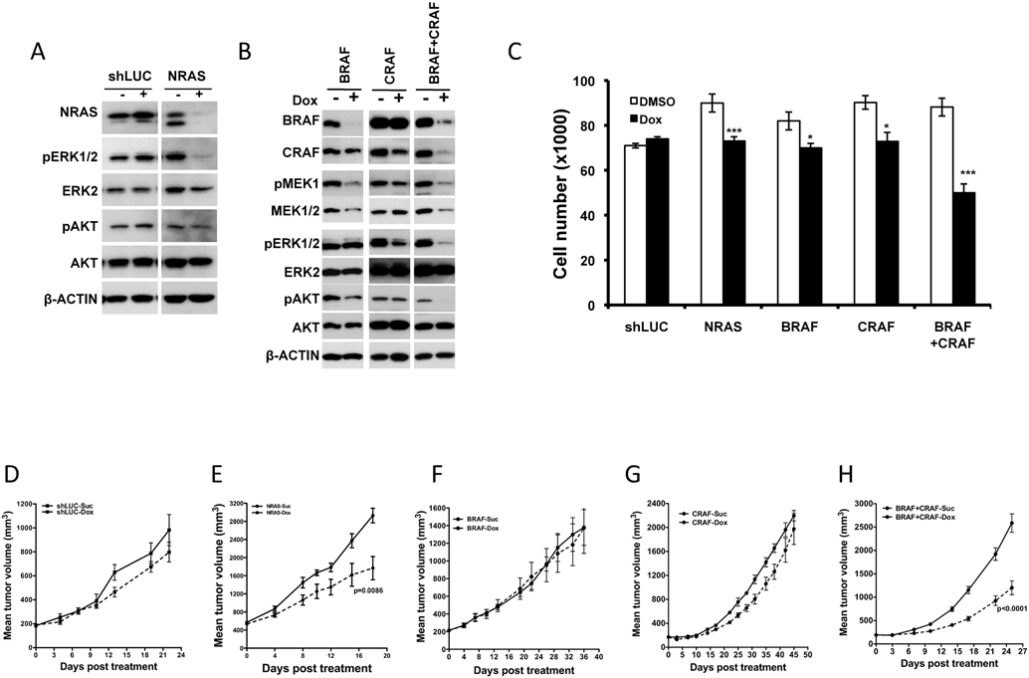
Combined targeting of BRAF and CRAF in NRAS^Q61K^-mutant SK-MEL-30 melanoma cell delays tumor formation *in vivo*. (A–B) Immunoblot analysis of SK-MEL-30 cells expressing control luciferase shRNA or shRNAs targeting NRAS, BRAF, CRAF or both BRAF and CRAF reveals respective protein knock-down and their effects on phosphorylation of downstream targets. (C) Proliferation of SK-MEL-30 cells expressing control luciferase shRNA or shRNAs targeting NRAS, BRAF, CRAF or both BRAF and CRAF. (D–H) shRNA targeting NRAS (E) or both BRAF and CRAF in SK-MEL-30 (H) delays tumor growth *in vivo*, whereas, induction of BRAF (F), CRAF (G) targeting shRNA or control luciferase (D) shRNA did not affect tumor growth. Each data point is the mean±SEM tumor volume of 10 mice. Dotted line in (D–H) represents data from dox treated animals.

**Figure 7:**
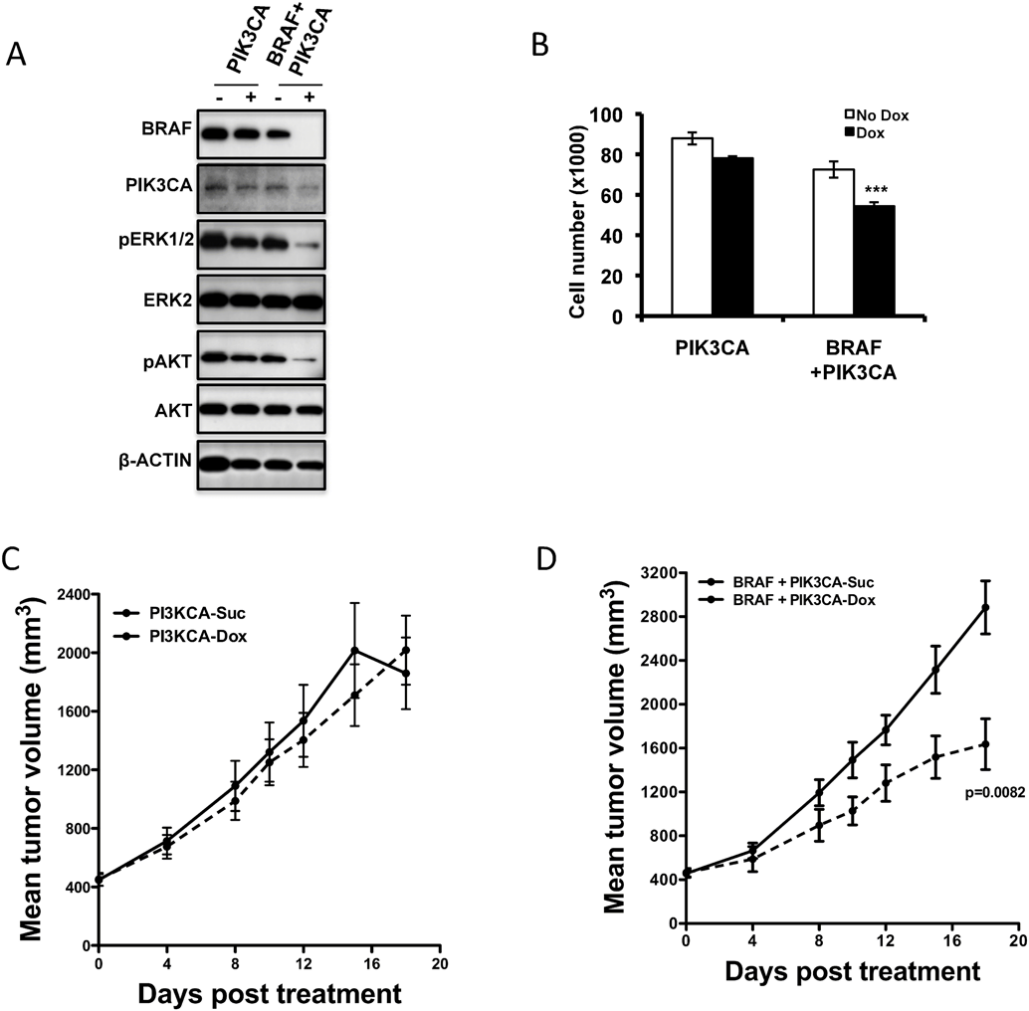
Combined targeting of PIK3CA and BRAF together in NRAS^Q61K^-mutant SK-MEL-30 melanoma cell delays tumor formation *in vivo*. (A) Immunoblot analysis of SK-MEL-30 cells expressing shRNAs targeting PIK3CA or both PIK3CA and BRAF reveal respective protein knock-down and their effects on phosphorylation of downstream targets. (B) Proliferation of SK-MEL-30 cells expressing control luciferase shRNA or shRNAs targeting PIK3CA or both PIK3CA and BRAF. (C–D) shRNA targeting both PIK3CA and BRAF in SK-MEL-30 (D) delays tumor growth *in vivo*, whereas, induction of PIK3CA (C) targeting shRNA alone did not affect tumor growth. Each data point is the mean±SEM tumor volume of 10 mice. Dotted line in (C–D) represents data from dox treated animals.

In order to understand if other NRAS mutant lines employ similar signaling cascades downstream of NRAS, we chose to study SK-MEL-30, another melanoma line with an NRAS^Q61K^ mutant allele. We generated SK-MEL-30 cells bearing inducible shRNA that target NRAS, BRAF, CRAF, PIK3CA alone, or combinations of BRAF and CRAF, or BRAF and PIK3CA. Inducible knock-down of NRAS, BRAF, CRAF individually or BRAF and CRAF or BRAF and PIK3CA together showed a decrease in the appropriate target protein with concomitant changes in downstream signaling as indicated by decreases in pERK and/or pAKT levels (Figure 6A, 6B, 7A). As with IPC298, knock-down of NRAS or the combined knock-down of BRAF and CRAF or BRAF and PI3KCA was effective in decreasing both the pERK and pAKT (Figure 6A, 6B, 7A). In *in vitro* proliferation studies, induction of shRNA targeting NRAS, BRAF, CRAF, dual targeting of BRAF/CRAF, or BRAF/PIK3CA had a reduction in proliferation of ∼20%, ∼12%, ∼20%, ∼40% and ∼28%, respectively (Figure. 6C and 7B), while the control shLUC or shRNAs targeting PIK3CA did not have a significant effect on proliferation compared to un-induced cells (Figure. 6C and 7B). In *in vivo* tumor growth studies, tumors carrying shRNAs that target NRAS alone, or combinations of BRAF and CRAF, or BRAF and PIK3CA, showed a significant delay in tumor growth (Figure 6E, 6H and 7D) compared to the corresponding sucrose controls. As observed in IPC298 cells, targeting BRAF, CRAF or PIK3CA individually in SK-MEL-30 cells did not effect in vivo tumor growth (Figure 6F, 6G and 7C). These results further confirm the requirement for sustained decrease in pERK and pAKT mediated signaling achieved either through combined targeting of BRAF and CRAF or BRAF and PIK3CA effector arms to be a more general requirement for effective therapy in NRAS mutant tumors.

### RAS-effector targeting promotes cell cycle arrest

**Figure 8:**
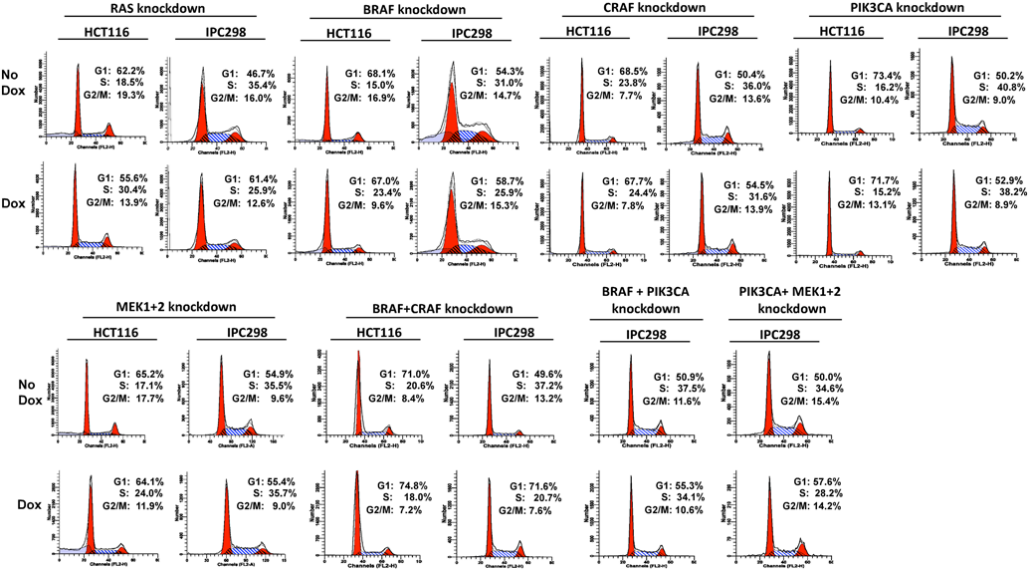
Targeting RAS and its downstream effectors leads to delay in cell cycle progression. A representative image showing the effect on cell cycle progression following knock-down of RAS and its downstream effectors as indicated in HCT116 or IPC298 cells. The complete results of cell cycle analysis from triplicates of two independent experiments along with the standard error of the mean values are presented in Table 1.

To address the mechanism by which loss of RAS (KRAS in HCT116 and NRAS in IPC298) reduced cellular proliferation and delayed tumor growth, we analyzed cell cycle progression and apoptosis induction in these cells following dox treatment. Induction of NRAS shRNA expression in the NRAS mutant-IPC298 line led to ∼65% of the cells arresting in G1 compared to ∼47% of the cells in G1 in untreated cells. This led to a delayed entry into S and G2/M phase (Table 1 and Figure 8). In the KRAS mutant HCT116 cells, KRAS knock-down following KRAS shRNA induction, lead to the cells accumulating in S phase, thereby preventing G2/M progression (Table 1 and Figure 8).

**Table 1:** Cell cycle status after RAS, RAF, PI3KCA and MEK knockdown.

Cell lines	G1 phase		S phase		G2 phase	
	No Dox	Dox	No Dox	Dox	No Dox	Dox
**HCT116-RAS**	63.59±0.9	55.30±0.9^**^	17.65±0.7	30.76±0.3^***^	18.77±0.3	13.94±0.6
**IPC298-NRAS**	47.18±0.9	65.14±1.9^***^	34.15±1.8	22.33±1.8^*^	18.67±1.8	12.53±0.1^*^
**HCT116-BRAF**	67.08±1.1	66.54±1.8	15.88±1.2	23.12±0.9^**^	17.05±0.1	10.34±0.9^**^
**IPC298-BRAF**	56.17±1.0	51.59±3.6	24.42±3.3	30.47±2.3	19.41±2.4	17.94±1.4
**HCT116-CRAF**	69.37±1.1	66.11±2.0	22.50±0.3	26.05±1.2^*^	8.13±0.8	7.84±0.9
**IPC298-CRAF**	50.29±1.1	55.05±1.4	34.98±0.6	31.17±0.2^*^	15.40±1.8	13.78±1.4
**HCT116-BRAF+CRAF**	70.92±0.5	74.59±0.9^*^	20.89±0.2	18.29±0.7^*^	8.18±0.4	7.12±0.2
**IPC298-BRAF+CRAF**	49.88±0.7	70.89±0.4^***^	38.85±0.3	21.41±0.4^***^	13.28±0.3	7.70±0.3^***^
**HCT116-PI3KCA**	74.06±0.8	71.65±1.2	16.02±0.4	15.73±0.3	9.91±0.4	12.61±0.4
**IPC298-PI3KCA**	49.89±1.6	51.99±0.7	39.93±0.8	38.64±0.3	10.18±1.0	9.37±0.5
**HCT116-MEK1+2**	63.59±0.9	55.30±0.9^**^	17.65±0.7	30.76±0.3^***^	18.77±0.3	13.94±0.6^**^
**IPC298-MEK1+2**	55.35±0.2	54.09±0.6	34.87±0.7	34.19±0.5	9.79±0.5	11.72±0.3
**IPC298-BRAF+PI3KCA**	51.18±0.4	55.54±1.3^*^	37.14±0.4	34.19±0.6^*^	11.67±0.4	10.27±0.7
**IPC298-PI3KCA+MEK1+2**	51.45±0.8	56.93±0.9^**^	32.70±1.3	29.85±0.9	14.52±1.8	13.92±0.3

*p<0.05, **p<0.01 and ***p<0.001 with respect to no dox control of same cell cycle phase.

Given that BRAF, CRAF, BRAF+CRAF and MEK1+2 double knock-down had an adverse effect on HCT116 cell proliferation and tumor growth, we examined the effect of targeting these genes on cell cycle progression. In all these knock-down cells, targeting the expression of these proteins led to an accumulation in G1 or S-phase, leading to delay in G2/M progression (Table 1 and Figure 8). These results suggest that targeting RAS and its downstream effector RAFs and the RAF-effectors MEK1+2 are mechanistically equivalent in KRAS mutant HCT116 cells. This is consistent with the fact that targeting RAS, BRAF, CRAF, BRAF+CRAF or MEK1+2 in HCT116 resulted in decreased proliferation *in vitro* and delayed tumor growth *in vivo*. Consistent with *in vitro* proliferation and *in vivo* tumor growth data, knock-down of BRAF, MEK1/2, and PIK3CA in IPC298 cells did not delay cell cycle progression (Table 1 and Figure 8). In contrast, knock-down of BRAF+CRAF, BRAF+PIK3CA, and MEK1/2+PIK3CA in IPC298 cells led to accumulation of cells in S or G1-phase consistent with the adverse effect on tumor growth observed *in vivo*.

To further dissect the mechanisms by which RAS targeting affects cell proliferation and delays tumor growth we tested the cells following RAS-targeting for apoptosis by annexin-V staining. As shown in Supplementary Figure 2, at the time-point observed, no increase in annexin-V staining was detected following RAS-knock-down in RAS-mutated IPC298 and HCT116 cells. However, BRAF knock-down in HCT116 showed a slight increase in annexin-V staining compared to the un-induced line, whereas all the other knock-down lines did not show an in increase in annexin-V staining following dox treatment. These data indicate that inhibition of cell proliferation and tumor growth upon RAS or a downstream RAS-effector knock-down is mainly due to delayed cell cycle progression.

## Discussion

Understanding the relative importance of oncogenic RAS effectors in tumor formation is fundamental to targeting RAS driven tumors using combinations of pharmacological inhibitors that are either available or in development. In this study we have addressed the relative importance of the RAS-effectors, RAF and PI3K, in a RAS mutant context in human tumor lines both *in vivo* and *in vitro* using a regulatable RNA interference system. We show that HCT116 colon tumor cells bearing an oncogenic KRAS^G13D^ mutation, IPC298 melanoma cells carrying an activated allele of NRAS^Q61L^ and SK-MEL-30 carrying a mutant NRAS^Q61K^ allele are dependent on the RAS pathway for their growth and proliferation both *in vitro* and *in vivo*. Knock-down of these RAS alleles *in vivo* leads to a significant delay in tumor growth and is consistent with the cell cycle arrest observed in the knock-down line *in vitro*. This is in agreement with a previous study in nude mice that demonstrated the requirement of mutant RAS for tumor formation, [16], [31]. To address the relevance of the effector arms of RAS in tumor maintenance and growth, we targeted the major RAS effectors RAF and PI3K using an inducible shRNA system. In HCT116, a colon cancer line, that carries a mutant KRAS allele, we find that interfering with the RAF effector axis at the level of RAF (BRAF and CRAF) and MEK (MEK1 and MEK2) was effective in delaying tumor growth. In contrast, there was no effect on tumor growth and maintenance when PIK3CA alone was knocked down, despite the fact that PIK3CA is mutated in this line. It is possible that in HCT116 cells, RAS could engage other PI3K family members to promote tumor growth. This is unlikely as pAKT levels are reduced following PIK3CA knock-down indicating that PIK3CA is the major PI3K isoform engaged by RAS in these cells. Further, this data suggest that the RAF-MEK-ERK cascade is predominantly engaged by KRAS in these cancer cells for growth and proliferation. In HCT116 cells, knock-in mutations that revert the mutant PIK3CA to WT affects it ability to proliferate, migrate and metastasize [9]. However, consistent with our *in vivo* data an HCT116 PIK3CA wild type line generated by knocking-in a copy of the WT PIK3CA in place of the mutant PIK3CA, did not block the knock-in revertant line's ability to form sub-cutaneous tumors [9]. Also, recently a study of colon cancer patients showed that PIK3CA mutations did not confer any significant effect on mortality among patients with KRAS-mutated tumors and is consistent with our findings on tumors growth in HCT116 cells [32]. These data taken together with our findings suggest that, in the colon cancer line, KRAS predominantly engages the RAF-MEK-ERK effector arm. This is in contrast to studies in other tumor types where the PIK3CA pathway is shown to be sufficient for tumor initiation and maintenance.

Our data shows that effective targeting of mutant NRAS driven tumor requires sustained reduction in pAKT and pERK. This can be achieved either by combined targeting of BRAF and CRAF, PIK3CA and BRAF or PIK3CA and MEK1+2. However, inhibition of MEK1+2 alone in the NRAS mutant line was not equivalent to inhibiting BRAF and CRAF together, as assessed by pERK and pAKT, suggesting the presence of differential signaling and feedback effects that arise from perturbing different nodes along the RAS/RAF signaling cascade. This is consistent with previously reported feedback and cross talk between the RAF and PI3K pathways [33]. These results have implications for therapeutic targeting of NRAS mutant melanomas where a dual BRAF/CRAF inhibitor or a BRAF and a PIK3CA inhibitor or a MEK and a PIK3CA inhibitor in combination is likely to show efficacy in the clinic. It is interesting to note that several studies also suggest the relevance of combined inhibition of CRAF and BRAF in a BRAF mutant context for efficacy and may prove to be relevant strategy for treatment of both NRAS and BRAF mutant melanomas [34], [35].

Our data suggests that the pathways engaged by mutant RAS that promote tumor formation show differences that are possibly inherent to each tissue or tumor type. Further investigation of the RAS-effector arms engaged within each tumor type may reveal differences that correlate with known tumor subtypes or form the basis for a better molecular classification of tumor subtypes. In the clinical context, understanding the specific effector arms engaged by RAS in driving tumorigenesis is critical to successfully treating these cancers. Developing gene signatures that allow identification and classification of tumors into RAS effector classes, based on effectors engaged by RAS, should allow for rational patient therapies that utilize appropriate pharmacological inhibitors either alone or in combination.

## Materials and Methods

### Cell lines and antibodies

Colon cancer cell line HCT116 was purchased from ATCC (American Type Culture Collection, Manassas, VA). IPC-298, a cutaneous melanoma line and SK-MEL-30, a melanoma line, were obtained from German Collection of Microorganisms and Cell Cultures (DMZ, Braunschweig, Germany). Mutation status of the cell lines used in this study is listed in Table-S1. Antibodies used in the study are as follows: CRAF, pAKT, AKT, ERK2, p-ERK1/2 (Thr202/Tyr204), MEK1, MEK2 and p-MEK1/MEK2 (Ser217/221) (Cell Signaling Technology, Beverly, MA); BRAF and NRAS (F-7; Santa Cruz Biotechnology, Santa Cruz, CA); KRAS (Novus Biologicals, Littleton, CO), PIK3CA (Millipore, Temecula, CA), β-actin (Sigma Life Science, St. Louis, MO); and horseradish peroxidase (HRP)–conjugated secondary antibodies (Pierce Biotechnology, Rockford, IL).

### Generation of inducible-shRNA cell pools

Hairpin oligonucleotides used in this study are listed in the Table-S2. They were based on a collection of siRNAs from Dharmacon Inc., (Chicago, IL) or based on previously published shRNAs [29], [30]. Inducible-shRNA bearing lentivirus constructs were made based on previously described methods [29], [30] by co-transfecting pHUSH-Lenti-GFP or pHUSH-Lenti-dsRed constructs containing a desired shRNA with plasmids expressing the vesicular stomatitis virus (VSV-G) envelope glycoprotein and HIV-1 packaging proteins (GAG-POL) in HEK293T cells using Lipofectamine (Invitrogen, Carlsbad, CA). Target cells were transduced with these viruses and sterile sorted (top 10%) by flow cytometry for presence of dsRed or GFP or both. Cells were characterized for knock-down by western blot analysis as previously described [29], [30].

### Anchorage independent growth assay

Twenty thousand cells in media containing 0.35% agarose with or without 1.0 µg/ml dox was plated on a layer of 0.5% agar in 10% serum. After a 2-week incubation at 37°C colonies were stained with 1 µM Calcein-AM, imaged using ImageXpress Micro (Molecular Devices, Sunnyvale, CA) and processed using MetaXpress Version 2.0 (Molecular Devices, Sunnyvale, CA) software to obtain colony counts.

### Xenograft studies

Six- to eight-week-old female athymic *nu/nu* mice were obtained from Charles River Laboratories (Wilmington, MA). For xenograft studies following trypsinization 5×10^6^ HCT116 cells or 1×10^7^ IPC298 or SK-MEL-30 were resuspended in 200 µl of PBS and injected into the right flank of nude mice. Once the tumors reached a mean volume of 150 to 200 mm^3^, the mice with similarly sized tumors were grouped and put on either 5% sucrose solution containing 1 mg/mL dox, for the treatment arm, or 5% sucrose only for the animals in control arm. Dox treatment induces the relevant shRNA. Tumor growth was monitored twice weekly for 4–8 weeks using calipers. Also, the mice were weighed twice a week. Mice whose tumor burden reached 2,000 mm^3^ were euthanized. Between 8 and 10 mice were used for each treatment group and results are presented as mean tumor volume±SEM.

### Cell Proliferation

In order to assess proliferation, 1×10^4^ cells/well in 100 µl volume was plated in a 96-well plate. Cells were treated with 1 µg/ml dox and proliferation was assessed after 4 days using Cell Titer-Glo Luminescent Cell Viability Assay kit (Promega Corporation, Madison, WI). A standard curve of luminescence as a function of cell number was used to calculate the number of cells in each treatment. Data are presented as mean±SEM from at least three replicate experiments.

### Cell cycle analysis

Cells were seeded in 6 well plates and treated with 1 µg/ml dox. After 72 h, cells were fixed in 70% ethanol for 1 hr at 4°C and stained with propidium iodide (PI) staining solution (50 µg/ml PI and 500 µg/ml DNase free RNase in PBS) and analyzed by flow cytometry (BD FACScan) using the CellQuest program. The data were analyzed using Mod Fit LT 3.0 cell cycle analysis software.

### Apoptosis assay

We used annexin V to measure the levels of apoptosis. Briefly, 4×10^5^ cells, RAS, BRAF and MEK1+2 shRNA containing HCT116 or IPC298 stable cells, were plated in 6-well plates and treated with dox (1 µg/ml for 72 hrs) in triplicate before staining with annexin V. After treatment cells were harvested, washed once with PBS, stained with FITC-conjugated anti-annexin V antibody and 100 µg/ml of propidium iodide in binding buffer for 15 min at room temperature. The cells were then analyzed with a flow cytometer (BD FACSCalibur). Background green fluorescence intensity due to presence of MEK1-shRNA-GFP in MEK1+2 cells were subtracted out before the calculating the fraction of annexin-V positive cells in case of MEK1+2 cells.

### Statistical Analysis

Student's t-test (two-tailed) was used for statistical analyses to compare treatment groups using GraphPad Prism 5.00 (GraphPad Software, San Diego, CA). A P-value <0.05 was considered statistically significant (*p<0.05, **p<0.01 and ***p<0.001).

## Supporting Information

Figure S1 MEK inhibitor is effective in KRAS mutant HCT116 cells. Treatment of HCT116 tumor-bearing mice with small molecule MEK inhibitor resulted in significant inhibition of tumor growth (p<0.001). Doses were well tolerated as determined by the change in animal weight (data not shown). MEK small molecule inhibitor was administered daily by oral gavage at a final concentration of 6 mg/kg. The compound was prepared fresh weekly in 0.5% methylcellulose and 0.2% Tween 80 in water and stored at 4°C. Tumor growth was monitored twice weekly for 4–8 weeks using calipers.
(0.02 MB TIF)

Figure S2 Induction of apoptosis measured by Annexin-V staining, in RAS mutant lines following induction of shRNA. Apoptosis in HCT116 or IPC298 lines expressing shRNAs that target KRAS or NRAS (A), BRAF (B) and both MEK1 and MEK2 (C). Results shown are mean±SEM of experiments from triplicates of two independent experiments. *P<0.001 vs untreated cells.
(0.14 MB TIF)

Table S1 (0.04 MB TIF)

Table S2 (0.21 MB TIF)
